# Hypoxic Hypoxia and Brain Function in Military Aviation: Basic Physiology and Applied Perspectives

**DOI:** 10.3389/fphys.2021.665821

**Published:** 2021-05-17

**Authors:** David M. Shaw, Gus Cabre, Nicholas Gant

**Affiliations:** ^1^Aviation Medicine Unit, Royal New Zealand Air Force Base Auckland, Auckland, New Zealand; ^2^School of Sport, Exercise and Nutrition, Massey University, Auckland, New Zealand; ^3^Department of Exercise Sciences, University of Auckland, Auckland, New Zealand

**Keywords:** oxygen, hypoxaemia, cognitive function, performance, safety

## Abstract

Acute hypobaric hypoxia (HH) is a major physiological threat during high-altitude flight and operations. In military aviation, although hypoxia-related fatalities are rare, incidences are common and are likely underreported. Hypoxia is a reduction in oxygen availability, which can impair brain function and performance of operational and safety-critical tasks. HH occurs at high altitude, due to the reduction in atmospheric oxygen pressure. This physiological state is also partially simulated in normobaric environments for training and research, by reducing the fraction of inspired oxygen to achieve comparable tissue oxygen saturation [normobaric hypoxia (NH)]. Hypoxia can occur in susceptible individuals below 10,000 ft (3,048 m) in unpressurised aircrafts and at higher altitudes in pressurised environments when life support systems malfunction or due to improper equipment use. Between 10,000 ft and 15,000 ft (4,572 m), brain function is mildly impaired and hypoxic symptoms are common, although both are often difficult to accurately quantify, which may partly be due to the effects of hypocapnia. Above 15,000 ft, brain function exponentially deteriorates with increasing altitude until loss of consciousness. The period of effective and safe performance of operational tasks following exposure to hypoxia is termed the time-of-useful-consciousness (TUC). Recovery of brain function following hypoxia may also lag beyond arterial reoxygenation and could be exacerbated by repeated hypoxic exposures or hyperoxic recovery. This review provides an overview of the basic physiology and implications of hypoxia for military aviation and discusses the utility of hypoxia recognition training.

## Introduction

Acute hypoxia is a major physiological threat during high-altitude flight and operations in military aviation. The human brain requires a continuous oxygen supply to function effectively. It is, therefore, vulnerable to environments with low atmospheric oxygen availability. At high-altitude, the reduced barometric pressure (hypobaria) lowers the partial pressure of inspired oxygen (PiO_2_) causing hypoxic hypoxia; henceforth referred to as hypoxia. The resulting hypoxaemia elicits a metabolic insult that impairs brain function and, with increasing severity, will cause loss of consciousness and eventually death. High-altitude experiments from balloon ascents in the 1800s were the first to describe the disastrous events of acute hypoxia (West, 2016). Then, during the Second World War in the 1930s and 1940s, it became apparent that the limitations of military aircraft were not necessarily due to mechanical or engineering failures, but the lack of oxygen to the brain of aircrew during flight. More recently, with the advent of pressurised environments and oxygen supply systems, pursuit into hypoxic environments is common. Acute hypoxia is the primary risk when life support systems malfunction in these environments and have been a focal area of research in military aviation for several decades.

Compared with civilian aviation, military aircrews have to navigate greater safety risks during flight, which increases the likelihood of being exposed to hypoxia. The onset of acute hypoxia can be rapid and pronounced (i.e., seconds) or slow and insidious (i.e., minutes-to-hours) depending on the type of equipment malfunction and magnitude of the hypoxic dose. Hypoxia may not present with clear physiological responses or perceptible signs and symptoms (Table 1), which is a major operational concern as unanticipated severe hypoxia will prevent recognition of hypoxia and implementation of emergency recovery procedures prior to loss of consciousness. For example, in a hypoxic emergency, pilots are required to immediately don oxygen mask, then to declare an in-flight emergency, descend below 10,000 ft, and land as soon as possible. The threat of hypoxia also extends to all aircrew, including rear crew, such as air warfare specialists, loadmasters, and medics. Whilst it is acknowledged that some military aircraft can elicit loading in the +Gz axis (i.e., commonly referred to as pulling Gs) to impair cerebral perfusion and cause stagnant hypoxia, such as high-performance jets, the physiological effects and mitigating factors markedly differ from hypoxic hypoxia and are considered outside the scope of the current review. Therefore, the aim of this review is to summarise the basic physiology of hypoxic hypoxia on brain function and recovery and to discuss the implications for military aviation, including the utility of hypoxia recognition training (HRT) for improving emergency responses to hypoxic incidences.

**Table 1:** Functional impairment and clinical status during hypoxia and hyperventilation-induced hypocapnia whilst sedentary at altitude.

Altitude		PaO_2_	PaCO_2_	Signs and symptoms	
ft	m	mmHg	mmHg	Hypoxia	Hypocapnia
0–5,000	0–1,524	80–95	40	No symptoms and normal function	No symptoms and normal function
5,000–10,000	1,524–3,048	80–60	35–40	Impaired performance of novel or highly complex tasks; and impaired night and colour vision	Minor hyperventilation and hypocapnia
10,000–15,000	3,048–4,572	40–60	30–35	Impaired performance of some simple tasks; further impairment of novel and complex tasks; mild hyperventilation; reduced physical capacity; and headache if exposure is prolonged	Mild dizziness; light-headedness; and feelings of unreality
15,000–20,000	4,572–6,096	30–40	25–30	Moderate-to-severe cognitive impairment; confusion; task fixation; impaired critical judgement; reduced willpower; impaired neuromuscular control; personality and mood changes (e.g., euphoria, pugnacious, morose, and aggressiveness); hyperventilation; visual impairments (including reduced peripheral vision, reduced light and colour intensity, and visual acuity); hot or cold flushes; sweating; central and peripheral cyanosis; impaired sense of touch and fine motor skills; sensory loss; nausea; fatigue; lethargy; and possible loss of consciousness	Moderate-to-severe dizziness; light-headedness; apprehension; neuromuscular irritability; paraesthesia of limbs and lips; tetany with carpopedal; and facial spasms
Above 20,000	Above 6,096	<30	<25	Myoclonic (muscle) twitches and convulsions; and loss of consciousness	Loss of consciousness may prevent worsening of hypocapnia

Adapted from previous publications (Gibson et al., 1981; Virués-Ortega et al., 2004; Gradwell and Rainford, 2016). *PaO_2_*, arterial blood oxygen partial pressure; *PaCO_2_*, arterial blood carbon dioxide partial pressure. Arterial blood gas tensions are estimates from the aforementioned studies and will vary based on duration of altitude exposure and the magnitude of the hypoxic ventilatory response. Signs and symptoms at lower altitudes are exacerbated with ascent, thus only novel signs and symptoms are stated within each higher altitude range; these can largely be classified into five categories: cognition, vision, psychomotor, psychological, and non-specific.

## Hypoxia in Military Aviation

Military aviation has the largest stake in hypoxia-related risks (Gradwell and Rainford, 2016). Since the early 1940s, aircraft have relied upon pressurised environments for safe, comfortable, and efficient flight at high altitudes as the atmospheric oxygen partial pressure (PO_2_) exponentially declines with increasing altitude [e.g., PO_2_ is 149 mmHg at sea-level and 49 mmHg at 25,000 ft (7,620 m)]. Although hypoxia-related aviation fatalities are rare, incidences are common, particularly in fighter and training aircraft (Cable, 2003; Files et al., 2005). For example, in 1055 aircraft depressurisation incidences between 1981 and 2003 within the United States Air Force, a reported 221 (21%) involved hypoxia, with three of these resulting in (preventable) death (Files et al., 2005). It is probable that hypoxia incidences are underreported, particularly when the onset of hypoxia is slow or gradual (i.e., insidious), suggesting the issue is greater than what is published.

The insidious onset of hypoxia may occur following an inboard leak within a pressurised cabin or when ascending in an unpressurised aircraft above 10,000 ft (3,048 m; PO_2_ < 100 mmHg). In such situations, hypoxia may not be identified as a causal factor for in-flight incidences and accidents. In contrast, rapid onset hypoxia may occur following a rapid depressurisation above 20,000 ft (6,096 m; PO_2_ < 63 mmHg), such as following an explosion or loss of the aircraft’s canopy. In these situations, environmental cues, physiological responses, and brain dysfunction are more evident and perceptible. Additionally, equipment malfunctions with oxygen supply systems, such as liquid oxygen systems or on board oxygen generating systems, are commonly reported, with the latter a concern in newer generation aircraft, such as the Hornet (The National Interest, 2016). Some individuals may also elicit mild symptoms and performance impairments at low altitudes (below 10,000 ft; Cable, 2003; Smith, 2005) and rapid loss of consciousness at moderate altitudes (e.g., 18,000 ft or 5,486 m; PO_2_ 70 mmHg; Chiang et al., 2012). Therefore, susceptibility to hypoxia may limit the operational capability for some military aircrew.

### Hypoxia

**Figure 1:**
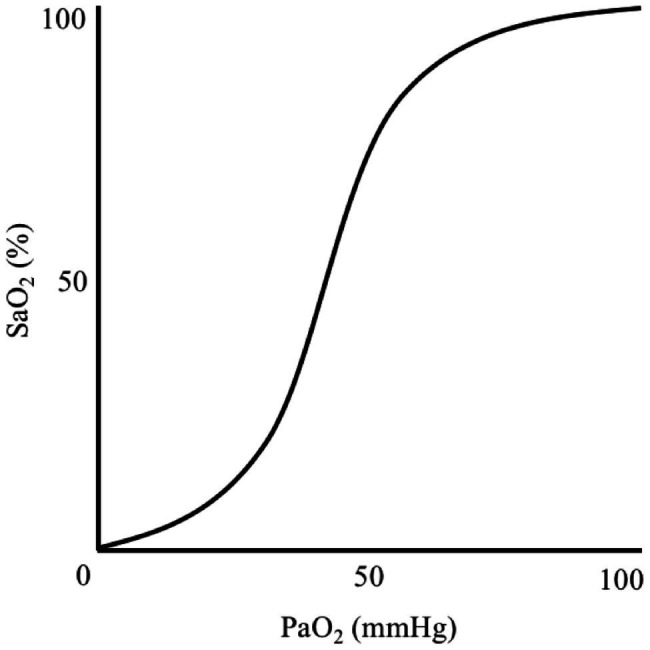
Illustration demonstrating the sigmoidal relationship between arterial blood oxygen-haemoglobin saturation (SaO_2_) and oxygen partial pressure (PaO_2_).

Generalised hypoxia is a state of insufficient oxygen availability throughout the body that is caused by exposure to a reduced atmospheric PO_2_, thus lowering PiO_2_ and disrupting the ventilation-perfusion equilibrium. A hypoxic cellular environment is caused by hypoxaemia, which is a reduction in arterial blood oxygen partial pressure (PaO_2_) and haemoglobin-bound oxygen saturation (SaO_2_) that results in inadequate oxygen delivery to tissues. Hypoxaemia is characterised by a sigmoidal relationship between PaO_2_ and SaO_2_, which occurs when breathing atmospheric PO_2_ below 149 mmHg (Adair, 1925; Lambertsen et al., 1952; Figure 1). In a resting, healthy individual at sea-level, an SaO_2_ is ~97–99% and remains relatively stable until PaO_2_ declines below ~80 mmHg (Collins et al., 2015). Nevertheless, humans can function, albeit impaired, with an SaO_2_ of 80–90% for hours-to-days as demonstrated in high-altitude, mountaineering studies.

The body’s initial compensatory responses to hypoxaemia involve an increase in cardiac output (Richardson et al., 1966) and stimulation of the ventilatory chemoreflex by the carotid bodies (Richard and Koehle, 2012). This cardiorespiratory upregulation aims to correct the ventilation-perfusion mismatch and increase arterial blood oxygenation (López-Barneo et al., 2016). Compensatory responses to hypoxaemia support cerebral oxygen delivery, including increased cerebral blood flow (CBF) to protect brain function (Friend et al., 2019). CBF increases when PaO_2_ declines below ~50 mmHg (i.e., ~85% SaO_2_) by 0.5–2.5% per 1% reduction in SaO_2_ (during isocapnic-hypoxia); however, this is not uniform for all brain regions (Ainslie et al., 2016; Hoiland et al., 2016). Ultimately, these compensatory mechanisms are insufficient and brain function deteriorates markedly during severe hypoxaemia with SaO_2_ declining below 50% prior to loss of consciousness in some individuals (Ernsting, 1963).

Signs and symptoms of hypoxia are common in most individuals following exposure to altitude, particularly above 10,000 ft (Table 1). These can largely be classified into five categories: cognition, vision, psychomotor, psychological (e.g., mood), and non-specific (Smith, 2008). The onset and intensity of hypoxic symptoms and compensatory responses depend on a variety of factors, including the altitude attained and the rate of ascent, PO_2_ of the breathing gas (if oxygen supply systems are in use), and duration of exposure. This hypoxic dose can be simulated in laboratory settings by manipulating the fraction of inspired oxygen (FiO_2_), barometric pressure, and duration of exposure (see Simulating hypoxic environments). However, hypoxic doses comprising different contributions of each factor do not necessarily elicit identical physiological effects (Conkin and Wessel, 2008; Conkin, 2016). The severity of hypoxia can be based on the level of blood or tissue oxygenation, or hypoxic signs and symptomology. There is large inter-individual variation in hypoxia tolerance, which may, in part, be attributable to the magnitude of the hypoxic ventilatory response and cardiovascular reflex (Virués-Ortega et al., 2004). These factors make comparisons between studies and interpreting their relevance to military aviation difficult.

### Hypocapnia

Hypocapnia tends to manifest following an increased ventilatory response to hypoxaemia and can elicit similar signs and symptoms as hypoxia (Bresseleers et al., 2010; Table 1). The interaction between PaO_2_ and arterial blood carbon dioxide partial pressure (PaCO_2_) are principle determinants of CBF (Hoiland et al., 2016), but not cardiac output (Richardson et al., 1966). Hypocapnia increases cerebral vasoconstriction to reduce CBF; whereas, both hypercapnia and hypoxia increase cerebral vasodilation and CBF (Willie et al., 2014). The brain is more sensitive to changes in PaCO_2_ than PaO_2_ (Kety and Schmidt, 1948; Willie et al., 2014), with CBF declining by ~3–4% per 1 mmHg reduction in PaCO_2_ (Brugniaux et al., 2007; Ainslie and Duffin, 2009; Willie et al., 2012). However, during severe hypoxaemia (i.e., <85% SaO_2_), PaO_2_ is the dominant influence on CBF (Kety and Schmidt, 1948). The initial responses to hypoxia may, therefore, be related to hypocapnia-induced (i.e., poikilocapnic hypoxia) impairment of CBF and cerebral oxygen saturation (ScO_2_; i.e., ischaemia and hypoxia; Virués-Ortega et al., 2004). This may be compounded by increased haemoglobin-oxygen affinity, which shifts the sigmoidal PaO_2_-SaO_2_ curve to the left (i.e., Bohr effect) and, therefore, increases haemoglobin-oxygen loading in the lungs and reduces oxygen unloading in the tissues, thus deceptively increasing SaO_2_ despite tissue hypoxia occurring.

When exposed to high altitudes, a greater tolerance to hyperventilation-induced hypocapnia can increase SaO_2_ and ScO_2_ (Ottestad et al., 2017). ScO_2_ is maintained by the net increase in CBF, which supplies higher oxygenated blood to maintain cerebral oxygen delivery (Willie et al., 2014). Nevertheless, maximising CBF (Cohen et al., 1967; Ogoh et al., 2013) and ScO_2_ (Dorp et al., 2007) during hypoxia appears to require maintenance of SaCO_2_ (i.e., isocapnic hypoxia) *via* CO_2_ administration. Poikilocapnic and isocapnic hypoxia may also elicit regional differences in CBF, with a previous study demonstrating blood flow in the internal carotid artery remaining unchanged and vertebral artery increasing during poikilocapnic hypoxia; whereas, both increased during isocapnic hypoxia (Ogoh et al., 2013). Therefore, when hypoxia-induced hyperventilation is sustained, the resulting hypocapnia appears to influence the effects of hypoxia.

### Simulating Hypoxic Environments

In training and research, hypoxia is induced using hypobaric and normobaric chambers or breathing systems. A combination of hypobaric and normobaric systems has also been employed, termed Combined-Altitude-Depleted-Oxygen (CADO; Singh et al., 2010). Hypobaric hypoxia (HH) reduces PiO_2_ due to a reduction in barometric pressure since the percentage of oxygen from sea-level to the limits of the troposphere remains ~21%. Whereas, normobaric hypoxia (NH) reduces PiO_2_ by decreasing the FiO_2_ with no change to barometric pressure. Therefore, an altitude of 25,000 ft can theoretically be simulated by breathing a gas mixture of 6.5% oxygen and 93.5% nitrogen at 760 mmHg (i.e., sea-level barometric pressure) assuming atmospheric water vapour partial pressure (PH_2_O) at 37°C is 47 mmHg; termed the “equivalent-air-altitude” (EAA). However, if PH_2_O is not accounted for in NH, the altitude would be underestimated, which increases in magnitude with ascent (Conkin, 2011). For example, dry NH equivalent to 25,000 ft (7,620 m) is actually 22,999 ft (7,010 m) once PH_2_O is accommodated. The EAA is employed to elicit a hypoxic dose to a specific altitude in order to induce isohypoxia (i.e., identical physiological responses, signs, and symptoms).

It should be emphasised that the physiological responses to HH and NH appear to differ, despite exposure to an identical PiO_2_, suggesting an independent effect of barometric pressure (Coppel et al., 2015; Millet and Debevec, 2021). This may be underpinned by duration of hypoxic exposure and increased physiological deadspace (i.e., the volume of inhaled air not included in gas exchange) in HH (Savourey et al., 2003). During brief (<5 min) exposure to very low PO_2_ (e.g., >25,000 ft), physiological differences between HH and NH are apparent, but minor, such as increased ventilation in NH and faster arterial blood deoxygenation in HH (Richard and Koehle, 2012). This may provide comparable hypoxic signs and symptoms as the body is unable to reach ventilation-perfusion equilibrium irrespective of barometric pressure (Richard and Koehle, 2012). Whereas, when hypoxic exposure is prolonged and gradual, physiological differences are more pronounced in HH compared with NH (Richard and Koehle, 2012; Debevec and Millet, 2014). For example, 40 min at 14,764 ft (4,500 m) elicited greater hypoxaemia, hypocapnia, blood alkalosis, and heart rate in HH compared with NH (Savourey et al., 2003). Moreover, during 30 min at 18,045 ft (5,500 m), participants reported more symptoms during HH than NH (Aebi et al., 2020b). Differing physiological responses between HH and NH may be partially explained by limitations of the alveolar gas equation (AGE), which is “PiO_2_ = (barometric pressure-PH_2_O) × FiO_2_” (Conkin and Wessel, 2008). The AGE assumes only FiO_2_ requires adjusting for each EAA to induce isohypoxia and neglects the (minor) influence of barometric pressure, which prevents exact interconversions between HH and NH.

Generally, NH models typically substitute oxygen for nitrogen to lower FiO_2_. Therefore, the gradient between alveolar and arterial nitrogen partial pressure is greater in HH compared with NH. This takes longer to reach a nitrogen equilibrium (i.e., nitrogen dilution or respiratory exchange effect), thus lowering alveolar PO_2_ and PCO_2_ (Conkin and Wessel, 2008). As such, the higher initial PaCO_2_ in NH increases ventilatory drive and CBF. Hypobaria also decreases air flow and work of breathing (Loeppky et al., 1997) and, due to greater physiological deadspace, increases the end-tidal PCO_2_/PaCO_2_ gradient, thus further attenuating the ventilatory response (Savourey et al., 2003). Hypobaria may also increase pulmonary vasoconstriction (Petrassi et al., 2012; Conkin, 2016) and, in contrast to the aforementioned findings, reduce CBF (Aebi et al., 2020a). Therefore, for each EAA, only PiO_2_ is equivalent between HH and NH; whereas, other factors contributing to the AGE may differ, such as gas flow distribution, diffusivity of gases and nitrogen kinetics, and the magnitude of hyperventilation-induced hypocapnia.

## Quantifying Hypoxia

### Measuring Hypoxaemia

Arterial blood oxygen partial pressure and SaO_2_ can be quantified directly using arterial blood gas co-oximetry. Whereas, peripheral blood oxygen saturation (SpO_2_) is an estimate of SaO_2_ that is measured indirectly using pulse oximetry. Pulse oximetry is based on photoplethysmography; an optical technique which illuminates the skin of the finger-tip, earlobe, or other tissue to measure changes in haemoglobin light absorption. Pulse oximetry is a non-invasive, immediate, and a convenient alternative to the gold standard, yet invasive, blood gas measurements (Mannheimer, 2007). A bias of below 3–4% between SaO_2_ and SpO_2_ is generally considered negligible for measurements under normoxic conditions (Nitzan et al., 2014), but when SpO_2_ is below 70–80%, the agreement with direct measures is reduced and the validity of SpO_2_ is compromised (Severinghaus et al., 1989). Under these conditions, there can be a systematic underestimation of SpO_2_ (Severinghaus et al., 1989; Ottestad et al., 2018); however, because pulse oximeters are typically not calibrated at these levels (Nitzan et al., 2014), the direction and magnitude of error are uncertain. Skin pigmentation, sex, and pulse oximeter design also increase SpO_2_ variability (Feiner et al., 2007).

### Measuring Cerebral Oxygenation

Measures of brain tissue oxygenation, such as ScO_2_, can provide more relevant and localised indices of oxygen deficit compared to systemic arterial blood gas measurements (i.e., SaO_2_ or SpO_2_). ScO_2_ can be measured directly using cerebral vessel blood sampling (Ernsting, 1963) and estimated non-invasively using near infrared spectroscopy (NIRS; Scheeren et al., 2012; Bickler et al., 2013). ScO_2_ measurements may be expressed relative to baseline or as absolute tissue saturation (MacLeod et al., 2012), which rely on proprietary algorithms (based on arterial and cerebral mixed venous haemoglobin-oxygen saturations) for their estimation, and can vary markedly. Moreover, skin pigmentation, sex, and NIRS design increase ScO_2_ variability (Bickler et al., 2013). This may underpin the inconsistent findings compared with arterial blood oxygenation following hypoxic exposure as ScO_2_ has been shown to decline to a similar (Ottestad et al., 2018), lower (Williams et al., 2019), and greater (Phillips et al., 2009) extent to SpO_2_.

### Field-Based Oximetry in Aviation

Currently, the prevalence of hypoxia incidences in aviation is based on self-reports due to an absence of biomonitoring. This makes it difficult to reliably state the contribution of hypoxia to flight safety events and to differentiate hypoxia from the effects hypocapnia or hypobaria (Ainslie et al., 2016; Hoiland et al., 2016). Whilst measurements of ScO_2_ have occurred within field studies of F-15 fighter pilots (Kobayashi et al., 2002), integration of NIRS, and other forms of oximetry, within aviation environments does not appear to be common practice. This is possibly due to difficulty integrating oximetry devices into aircrew flight clothing and equipment and ascertaining reliable measurements, which may be exacerbated by additional factors pertinent to aviation (Phillips et al., 2012), such as changes in barometric pressure, gravitational forces, human movement, and perspiration.

## Hypoxia, Brain Function, and Performance

### Metabolic Vulnerability of the Brain

The brain’s obligatory demand for oxygen and reliance on oxidative energy metabolism makes it vulnerable to oxygen deficit. Despite weighing ~2% of body mass, the brain requires 20–25% of the body’s resting energy requirements, resulting in an oxygen consumption per unit of mass greater than all other tissues (Bailey, 2018). The majority of the brain’s energy requirement supports neuronal signalling, involving networks composed of billions of neurons, with 40–60% of the energy contributing toward driving ions up gradients (Bailey, 2018). During hypoxia, cerebral oxygen consumption appears to marginally increase, or at least remain similar to normoxic conditions (Ainslie et al., 2016), to maintain adequate rates of oxidative energy metabolism. This compensatory effect suggests energy production is not always impaired, at least when hypoxia is not severe. Rather, under these circumstances, hypoxia may impair the metabolism of neurotransmitters (Gibson et al., 1981), although impairment to other metabolic factors is likely. These derangements in cerebral metabolism can be detected by electrophysiological markers, such as EEG (Kraaier et al., 1988; Malle et al., 2016; Altbäcker et al., 2019; Rice et al., 2019), particularly at a SaO_2_ of ≤75% or PaO_2_ of ≤40 mmHg (Goodall et al., 2014). Nevertheless, simultaneous performance of cognitive tasks may negate reductions in EEG power (Malle et al., 2016), which would make it difficult to evaluate the magnitude of impairment to hypoxia-induced cerebral metabolism in operational environments, such as when piloting an aircraft.

### Brain Injury

Humans appear remarkably tolerant to hypoxia (Bailey et al., 2017; Bailey, 2019); however, the harmful effect of repeated exposures remains uncertain. In fact, some researchers suggest there are no long-lasting detriments following hypoxia, unless perfusion is impaired (i.e., ischaemia; Bickler et al., 2017), as hypoxia-ischaemia produces more severe effects on the brain (Lee et al., 2000). For example, in a population of breath-hold divers regularly experiencing hypoxaemia below an SpO_2_ of 60%, cognitive performance appeared normal (Ridgway and McFarland, 2006), which was interpreted as the absence of hypoxic brain injury in a recent review (Bickler et al., 2017). Nevertheless, if hypoxia becomes sufficiently severe, ischaemia may result. Neuronal tolerance to hypoxia may also be greater than initially thought (Bailey, 2019); for example, bioenergenic reserves may be sufficient for ~3–4 min following withdrawal from lifesaving therapy in brain injured patients (Dreier et al., 2018). Further research is required to better understand the impact of hypoxia and hypoxia-induced ischaemia on brain injury and its operational significance for exposure envelopes experienced by aircrew. It should also be noted here that non-hypoxic dysbaric neurological injuries can result from hypobaria *per se*. The most common acute condition being neurological decompression sickness (Jersey et al., 2010; Vann et al., 2011; Hundemer et al., 2012). It is also emerging that hypobaria may independently influence neuroinflammatory responses (Tchantchou et al., 2021) and induce symptoms associated with acute mountain sickness or high-altitude cerebral oedema (Basnyat and Murdoch, 2003), and during chronic/career exposure alter white matter integrity (McGuire et al., 2014, 2019). The pathophysiology of these conditions is poorly understood.

### Cognition

Hypoxia impairs a spectrum of cognitive domains as previously described in narrative (Virués-Ortega et al., 2004; Petrassi et al., 2012; Yan, 2014; Taylor et al., 2016) and systematic reviews (McMorris et al., 2017). Both simple (e.g., simple and choice reaction speed) and complex (e.g., processing speed, working memory, short-term memory, attention, executive function, and novel task learning) tasks are negatively affected by hypoxia; the degree of which can vary greatly between individuals (McMorris et al., 2017). Given the dynamic environment of military aviation, even small impairments to cognition may result in a serious or fatal accident. Despite the physiological differences induced by NH and HH, only slight differences in cognitive impairment may be attributed to dysbaria (Aebi et al., 2020b). Moreover, whilst it is possible that repeated exposure to hypobaria resulting in loss of white matter integrity can impair cognition (McGuire et al., 2014), hypoxia itself is largely regarded as a greater acute threat to cognition.

Previous research has aimed to categorise altitudes that impair specific domains of cognitive function (Fowler et al., 1987). Generally, at high-altitudes, particularly above 15,000 ft (4,472 m; Petrassi et al., 2012), or with lower arterial blood oxygenation (Ochi et al., 2018; Williams et al., 2019) or cerebral oxygenation (Williams et al., 2019), there is greater and more predictable impairment to cognition. Complex and novel cognitive task performance may be impaired between 6,500 and 12,000 ft, which typically invoke an SpO_2_ of 70–90% (Legg et al., 2012, 2014; Petrassi et al., 2012; Pilmanis et al., 2016). Whereas, simple cognitive task performance (e.g., card naming and/or sorting) may not deteriorate until below an SpO_2_ of 65% (Hoffman et al., 1946; Mitchell et al., 2019), which typically occurs following exposure above 18,000–25,000 ft. Although the relevance of these cognitive deficits to military aviation is difficult to interpret, operational tasks have been impaired by hypoxia, such as simulated flight performance (Temme et al., 2010; Robinson et al., 2018).

The severity of hypoxia at which meaningful cognitive impairment begins is uncertain. Complex, compared with simple, cognitive tasks appear more sensitive to hypoxia, such as central executive function (McMorris et al., 2017), presumably due to increased oxygen demand of greater neural activation (Raichle and Gusnard, 2002). However, complex tasks vary in sensitivity (Williams et al., 2019). It is also possible that more complex tasks protect against the detrimental effects of hypoxia (Malle et al., 2016), potentially by eliciting a compensatory cerebral autoregulatory response. Simple cognitive tasks, such as simple and choice reaction speed, may also be impaired (Friend et al., 2019) or maintained (Williams et al., 2019) during hypoxia (75–80% SpO_2_). These inconsistencies may have been due to underlying physiological differences between studies, such as regional CBF and ScO_2_. It should also be noted that the preservation of cognitive performance, such as speed, may be at the expense of accuracy, or vice versa (Friend et al., 2019; Williams et al., 2019).

Most studies examining the effect of hypoxia on cognitive performance have employed single bouts of hypoxia at a fixed altitude or EAA. This approach may not accurately reflect hypoxia doses encountered in real-world scenarios. For example, a pilot may experience moderate-to-severe hypoxia at a high altitude followed by mild hypoxia once they descend to a lower altitude. In a recent study, flight performance deteriorated during exposure to simulated 10,000 ft preceded by exposure to 25,000 ft (Robinson et al., 2018), which suggests a lagging effect or an interaction of the two hypoxic exposures, despite the absence of hypoxaemia. This effect of sequential hypoxic exposures with varying recovery times on cognitive performance is yet to be fully elucidated (discussed below), but is critical if real-world operations are to continue following recovery from hypoxia. Existing research also does not adequately address the interaction of additional real-world scenarios on cognition, such as reduced cerebral perfusion following the onset +Gz forces and or rapid changes in barometric pressure (e.g., rapid or explosive depressurisation).

Arterial blood carbon dioxide partial pressure and/or acid-base status (i.e., alkalosis) not only influences cerebrovascular haemodynamics, but also cognitive performance (Leacy et al., 2019). For example, a recent study demonstrated hyperventilation-induced hypocapnia (~60–80 min) slowed simple and choice reaction time during both normoxia (end-tidal CO_2_ of ~33 mmHg) and hypoxia (end-tidal CO_2_ of ~38 mmHg), with no differences between conditions (Friend et al., 2019), suggesting an independent effect of hypocapnia on hypoxia-induced cognitive dysfunction. This may partly explained by lower CBF in the poikilocapnic compared with isocapnic hypoxia condition; however, the increased CBF had no effect on ScO_2_ (Friend et al., 2019). Overall, this corroborates previous research demonstrating that supplementing with CO_2_ during hypoxia (80% SpO_2_) can mitigate performance impairments of complex cognitive tasks (Dorp et al., 2007). Therefore, it is important to distinguish the influence of hypoxia and hypocapnia, including differences in regional brain blood flow and oxygenation, on cognitive impairment and its implications for military aviation.

Recognising hypoxia before profound cognitive impairment is critical for implementing emergency recovery procedures. Since hypoxia impairs the ability to identify cognitive impairment within oneself (Mitchell et al., 2019), the capacity to recognise hypoxic symptoms is also compromised (Asmaro et al., 2013; Rice et al., 2019). Moreover, hypoxia can be insidious and include pleasant sensations, such as euphoria, decreased inhibitions, and a strong sense of wellbeing, which will attenuate any perception of urgency. For example, in a recent study, more than 20% of participants did not action emergency procedures during hypoxia and 17% actioned emergency procedures without being hypoxic, meaning 37% of participants either misidentified or failed to recognise they were hypoxic (Rice et al., 2019). Whilst it is possible to perceive and recognise hypoxic symptoms prior to cognitive impairment (Turner et al., 2015; Pilmanis et al., 2016), this may not occur for all individuals. Measuring lapses in cognitive performance, rather than average performance, could also increase the sensitivity of tests as increased effort may mask potential decrements (Phillips et al., 2016). It should also be noted that increased mental effort and task-fixation, a common sign of hypoxia, to maintain cognitive performance of operational tasks may detract from recognizing hypoxic symptoms.

## Time-Of-Useful-Consciousness

**Figure 2:**
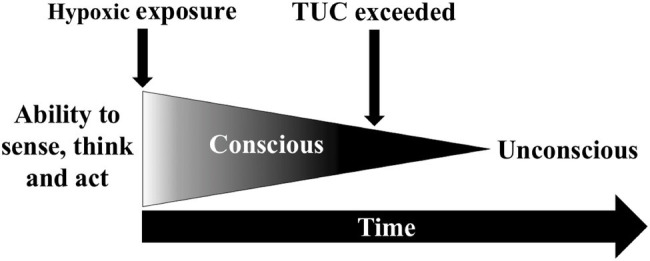
Time-of-useful-consciousness (TUC) paradigm.

If arterial and tissue deoxygenation does not stabilise, brain function progressively declines, which occur exponentially at a very low PiO_2_. The initial phase is referred to as the time-of-useful-consciousness (TUC) and is the duration of *effective and safe performance of operational tasks*, which is followed by mental confusion and unconsciousness (Figure 2; Hoffman et al., 1946; Hall, 1949). The validity of the TUC criterion has been debated since its inception (Hoffman et al., 1946; Izraeli et al., 1988) as TUC endpoints differ between studies and often fail to reflect the demands of operational environments. These endpoint tasks have included: card sorting (Hoffman et al., 1946), card recognition (Mitchell et al., 2019), single and choice reaction speed (Hall, 1949), two-digit number addition (Izraeli et al., 1988), sequential numeric writing (Yoneda et al., 2000), handwriting (Yoneda and Watanabe, 1997), behavioural disturbances (Malle et al., 2016), and the magnitude of hypoxaemia (Hoffman et al., 1946; Malle et al., 2016). Whether these provide an accurate estimate of time to recognise hypoxia and implement emergency recovery procedures is uncertain. The reduced reliability of pulse oximeters in very low SpO_2_ ranges (Severinghaus et al., 1989) also has potential to confound the estimation of hypoxaemia during TUC protocols.

Table 2 summarises the estimated TUC ranges at various altitudes and EAAs. For example, TUC is 3–5 min at 25,000 ft (PO_2_ 49 mmHg) and declines to less than 15 s at 50,000 ft (PO_2_ 8 mmHg). With more complex tasks, such as self-directed recovery, TUC may be shorter than current estimates, particularly for altitudes below 35,000 ft (PO_2_ <28 mmHg; Phillips et al., 2016). In operational environments, it is also unlikely that the full TUC will be available to implement emergency recovery procedures as hypoxia is likely to be recognised after hypoxaemia occurs (i.e., SpO_2_ is <80%; Varis et al., 2019; Leinonen et al., 2020). For example, in Hawk pilots, hypoxia was first recognised with an SpO_2_ of ~75% (Varis et al., 2019). Further research investigating TUC should, therefore, revaluate current durations specific to real-world tasks and use different subgroups within military aviation to ascertain if there are differences depending on the role within an aircraft (e.g., pilot vs. rear crew) as current values may be an overestimation. It could be difficult to achieve this, as temporal performance measurements must be ascertained in seconds (not minutes) since brain function deteriorates rapidly during severe hypoxia.

**Table 2:** Estimated TUC with increasing hypoxia severity.

Altitude		PiO_2_	EAA FiO_2_	SpO_2_	TUC	
ft	m	mmHg	%	%	Standard	RD
0	0	149	20.9	97–99	Unlimited	Unlimited
5,000	1,524	122	16.1	90–95	Unlimited	Unlimited
10,000	3,048	99	13.1	85–95	Unlimited	Unlimited
15,000	4,572	80	10.5	70–85	Hours	Hours
18,000	5,486	70	9.2	60–70	20–30 min	10–15 min
22,000	6,706	57	7.5	<70	10 min	5–6 min
25,000	7,620	49	6.5	<65	3–5 min	1.5–2.5 min
28,000	8,534	42	5.5	<60	2.5–3 min	1–1.5 min
30,000	9,144	37	4.9	<55	1–2 min	0.5–1 min
35,000	10,668	28	3.6	<50	0.5–1 min	0–15 s
40,000	12,192	20	2.6		15–20 s	Nominal
43,000	13,107	16	2.1		9–12 s	Nominal
50,000	15,240	8	1.0		<12 s	Nominal

Adapted from previous publications (Gibson et al., 1981; Virués-Ortega et al., 2004; Gradwell and Rainford, 2016; Phillips et al., 2016). *PiO_2_*, inspired oxygen partial pressure; *EAA*, equivalent-air-altitude; *FiO_2_*, fraction of inspired oxygen; *SpO_2_*, peripheral oxygen saturation; *TUC*, time-of-useful-consciousness; and *RD*, rapid decompression. FiO_2_ is calculated based on 760 mmHg sea-level pressure and assuming a partial pressure of water vapour at 37°C is 47 mmHg. SpO_2_ ranges are estimates for exposure following the minimum of the TUC range based on the aforementioned publications and the authors’ observations of individuals exposed to these altitudes.

Time-of-useful-consciousness estimates for altitudes below 35,000 ft are characterised by large ranges due to inter-individual variability in hypoxia (and hypocapnia) tolerance. Some of the lowest tolerable levels of hypoxaemia also appear to be from opposite ends of the atmospheric-biospheric pressure system (Bailey et al., 2017): PaO_2_ of 19 mmHg (SaO_2_ 34%; PaCO_2_ 16 mmHg) in an altitude acclimatised mountaineer on descent from the summit of Mount Everest (Grocott et al., 2009); PaO_2_ of 22.5 mmHg (SaO_2_ 48%; PaCO_2_ 29 mmHg) during simulated descent from 30,000 ft in a high-altitude parachutist (Ottestad et al., 2017); and PaO_2_ of 23 mmHg (SaO_2_ 38%; PaCO_2_ 61 mmHg) in a free-diver following static apnoea (Bailey et al., 2017). TUC may also be extended by: (1) oxygen pre-breathing (Malle et al., 2016), which increases oxygen stores in the lungs (Tanoubi et al., 2009); (2) greater haemoglobin oxygen carrying capacity of the blood (Hall, 1949); and (3) avoiding physical activity during exposure to hypoxia (Busby et al., 1976). Nevertheless, TUC does not appear to be extended by previous hypoxia exposures, suggesting it is not trainable (Izraeli et al., 1988; Mitchell et al., 2019).

## Recovery of Brain Function Following Hypoxia

Cognitive impairment may persist for several minutes-to-hours following arterial blood reoxygenation (Phillips et al., 2009, 2015; Beer et al., 2017; Varis et al., 2019). For example, after 10 min normobaric hypoxia (simulated 20,000 ft), reaction times were impaired during a 10-min recovery (normoxic) period, despite resolution of hypoxaemia within ~1 min (Phillips et al., 2009). This was suggested to be due to poor cerebral reoxygenation (Phillips et al., 2009), which was also demonstrated in follow-up study for up to 24 h following 30 min normobaric hypoxia (simulated 18,000 ft), which occurred alongside impaired simple and choice reaction speed (Phillips et al., 2015); therefore, the brain may reoxygenate at a slower rate to peripheral tissue. This means that performance of operational tasks or implementation of emergency recovery procedures may continue to be compromised following apparent recovery from hypoxia. This “hypoxia hangover” was demonstrated in a group of experienced Hawk pilots, demonstrating impaired (simulated) flight performance 10 min after recovery with 100% oxygen following a hypoxic exposure (~75% SpO_2_; Varis et al., 2019), which emphasies the need to land as soon as possible following hypoxic recovery. Nevertheless, not all studies demonstrate delayed cerebral reoxygenation (Uchida et al., 2020). Further research is required to determine whether there are operationally relevant temporal effects on brain function and cognitive performance following recovery from hypoxia.

### Hyperoxic Recovery

Breathing air comprising more than 21% oxygen (i.e., hyperoxia) to accelerate recovery from hypoxia is a common practice in military aviation. There is cause for enquiry whether hypoxia proceeded by hyperoxia breathing is harmful to the brain given recovery from ischaemic-hypoxia using more than 21% oxygen can cause brain injury (Shimabuku et al., 2005; Koch et al., 2008; Chalkias and Xanthos, 2012). Some individuals may also experience a transient (15–60 s) worsening of hypoxic symptoms and brain dysfunction during sudden reoxygenation of arterial blood, which is referred to as the *Oxygen Paradox* (Latham, 1951). This may be underpinned by hypoxia-induced hypocapnia and reduction in peripheral vasoconstriction, causing cerebral vasoconstriction and hypoperfusion (Gradwell and Rainford, 2016). For example, although 100% oxygen breathing following acute NH increased arterial blood reoxygenation faster than room air (i.e., 21% oxygen), recovery was associated with a robust EEG slowing and impaired working memory (Malle et al., 2016). This suggests breathing hyperoxic air following hypoxia may be more hazardous than normoxic recovery, which may impact performance of operational and safety-critical tasks. Further research investigating the effects of hypoxic recovery by breathing varying PO_2_ levels and how this differs with hypoxia severity is required. Moreover, the effects of CO_2_ inclusion in recovery gases should also be explored.

## Training and Preparing For Hypoxic Incidents

Hypoxia recognition training is a critical component of military aviation training of aircrew (Neuhaus and Hinkelbein, 2014) and could have implications in other operational environments. Currently, the North Atlantic Treaty Organisation Standardisation Agreement (STANAG) and Air Force Interoperability Council recommend refresher training a maximum of every 5 years; however, some countries may require more frequent trainings for at-risk aircrew. The primary rationale for HRT is the intentional induction of hypoxia within a safe and controlled environment to: (1) familiarise individuals with their constellation of personal hypoxia symptoms, including order of appearance and intensity; (2) experience the speed of onset and insidious nature of hypoxia; (3) observe hypoxia-induced cognitive and psychomotor impairment in others; and (4) practice using equipment and implementing emergency recovery procedures. An individual’s *most prominent* symptoms are reported to be consistent for up to 4–5 years for a given hypoxic dose (Woodrow et al., 2011; Johnston et al., 2012; Tu et al., 2020), which is referred to as their *Hypoxic Signature* (Smith, 2008). However, not all individuals accurately remember symptoms following hypoxia exposures in training (Smith, 2008; Woodrow et al., 2011; Tu et al., 2020) and operational (Files et al., 2005) environments. Reported hypoxic symptoms during training may also be different to operational environments, which could be due to a reduced capacity for memory recall, as well as differences in hypoxic dose, environmental conditions and biological variation.

Currently, there is a scarcity of research evaluating the efficacy of HRT and how it translates to hypoxia recognition in operational environments. Nevertheless, numerous anecdotal reports highlight the importance of HRT for improving operational safety (Cable, 2003; Files et al., 2005). Mask-on normobaric HRT was reported to reduce the time to recognise hypoxia in 64% of participants (Leinonen et al., 2020); however, there was no control group and the operational experience of participants between HRT sessions (i.e., ~2.4 years) could have interfered with the effect of HRT. Further, it would seem prudent to isolate how HRT could benefit a greater proportion of individuals to recognise hypoxia, which may warrant individualised approaches to HRT. The threshold at which hypoxaemia should reach during HRT should also be evaluated due to impairments on learning and memory (Nation et al., 2017), which would be counterintuitive to the aim of the training. Generally, pulse oximetry appears to be the preferred method determining hypoxia during HRT, with profiles being terminated when SpO_2_ declines below ~65–70%. The interaction of other physiological stressors, such as fatigue, temperature, and dehydration, on hypoxia recognition is also unknown, which is relevant given their prevalence within military aviation.

Although hypobaric chambers provided the initial tools to induce hypoxia, reduced oxygen breathing devices (ROBD) providing mask-on NH (Sausen et al., 2001) and a combination of HH and mask-on NH (i.e., CADO; Singh et al., 2010) have more recently been incorporated into HRT to prevent potential adverse effects of barometric pressure reduction, such as decompression sickness (Webb and Pilmanis, 2011), white matter hyperintensities (McGuire et al., 2014, 2019; Sherman and Sladky, 2018) and barotrauma. Additional advantages of ROBDs are their simplicity, ease of transport, reduced expense, and lower maintenance, and they can be the preferred mode of HRT for some individuals, particularly fighter pilots (Artino et al., 2006). NH using the ROBD is purported to closely replicate symptoms experienced within hypobaric chambers for brief exposures (Self et al., 2011); however, this remains controversial and the ROBD may not necessarily mirror hypoxic symptoms experienced by aircrew in operational environments (Deussing et al., 2011). Moreover, issues with breathing-gas flow rates when using the ROBD may alter hypoxia symptoms, particularly air hunger (Artino et al., 2009). Physiological differences between HH, NH, and CADO are suggested to be irrelevant to symptomology (Singh et al., 2010) and, thus, provide equivalent training value. However, this does not necessarily hold true (Aebi et al., 2020b) because hypocapnia recognition (hyperventilation-induced) may not be accounted for, and can be the primary indication of a hypoxic environment at moderate altitudes (Petrassi et al., 2012).

Hypoxia recognition training should include a high level of fidelity, with signs and symptoms of hypoxia reflecting what is likely to be experienced within operational environments. Hypoxic exposures should, therefore, require individuals to perform cognitive tasks specific to the aims of the training session. The ROBD allows individuals to engage in a variety of operational-specific tasks without restriction from the confines of the chamber and changes in pressure. For example, tactical flight simulation enables decision-making training, implementation of actual emergency recovery procedures, and the continuation of the hypoxia training mission until simulated landing. Alternatively, the hypobaric chamber provides a group environment for hypoxia to be viewed in others and barometric pressure changes (i.e., gradual and rapid decompression), which can elicit important signs and symptoms for recognising hypoxia, such as ear popping. If task saturation occurs using either the hypobaric chamber or ROBD, the subtle signs and symptoms are less likely to be perceived; therefore, depending on the training aim, this can either compromise or enhance HRT. A targeted variety of HRT methods and hypoxic doses, or customised approach, will best prepare individuals to recognise the hypoxia symptoms they are likely to experience in operational environments.

## Conclusions and Future Directions

Hypoxia is a major physiological threat during high-altitude flight and operations in military aviation. The extent of the issue is probably underestimated due to a lack of rigorous biomonitoring of military aircrew (i.e., pilots and rear crew). Reducing the risk of hypoxic-related incidents and accidents requires oxygen supply systems, pressurised environments, HRT, implementation of emergency recovery procedures, and adherence to safety regulations (e.g., mask wearing); however, there is a risk of malfunction for all life support systems, improper use of equipment, and failure to adhere to safety regulations. The ensuing hypoxia can present within seconds-to-minutes, such as a sudden abrogation of oxygen supply (e.g., failure of oxygen supply system), or develop gradually over minutes-to-hours, such as a slow decompression within the aircraft. Even minor impairments to brain function resulting from hypoxaemia, cerebral hypoxia, or hyperventilation-induced hypocapnia can be catastrophic in military aviation due to the dynamic and demanding environment aircrew must operate within. However, the relevance of hypoxia-induced brain dysfunction for military aviaition can be difficult to accurately quantify, particularly due to the large inter-individual variation in hypoxia tolerance and concurrent effects of hypoxia-induced hypocapnia.

Wearable biomonitoring can be used to signal the early stages of hypoxia or hypocapnia. Measures of SpO_2_, SaO_2_, SaCO_2_, and ScO_2_ could warn aircrew prior to brain function diminishing below recoverable levels. Numerous technological devices are available that continuously monitor oxygenation (e.g., pulse oximetry and NIRS); however, the measurement of carbon dioxide within the body is less common. Potentially, inbuilt breath-by-breath gas analysers within breathing masks may provide insight into arterial blood gas levels, *via* end-tidal PO_2_ and PCO_2_ measurement. Moreover, validated EEG techniques measuring real-time brain wave activity may also be able indicate hypoxia-induced brain dysfunction. These devices appear to be rarely used within military aviation, which could be due to difficulty incorporating them into the life support equipment and acquiring accurate measurements within extreme environments (e.g., high gravitational forces during fighter jet manoeuvres or hypobaria). If successful, monitoring of physiological status would mean that hypoxia and hypocapnia are reported more often and accurately, thus improving the surveillance of operational hypoxic and hypocapnic events. It would also provide reassurance on proper treatment procedures as hypoxia and hypocapnia can present similarly.

Hypoxia tolerance varies markedly between individuals. Arterial and cerebral oxygenation, CBF, and ventilatory responses can all vary greatly to a specific hypoxic dose, which may underpin differences in simple and complex cognitive outcomes. Importantly, simple cognitive tasks are unlikely to correlate well with the requirements of real-world emergencies, but may provide a reliable surrogate for automated operational tasks. It is, therefore, recommended to use a range of cognitive tests when examining the effects of hypoxia on cognition, particularly complex tasks requiring executive, innovative, creative, and flexible thinking. These domains are necessary for comprehending and functioning within real-world, novel, and dangerous scenarios that demand situational awareness, complex multi-tasking, self-reflection, effective communication, managing behaviours and emotions, evaluating evolving situations, and decision making. Further, if complex tasks have increased oxygen demand, the requirement for supplemental oxygen should not solely be based on altitude or hypoxaemia, but also operational tasks. Recovery of brain function following hypoxia should also be assessed as there appears to be a lagging effect, despite resolution of hypoxaemia, which may differ based on the level of oxygen administered and inclusion of carbon dioxide.

Hypoxia recognition training appears to be an important safety precaution to prevent hypoxic fatalities by enhancing the response to unanticipated hypoxia. Although the efficacy of HRT is yet to be systematically evaluated, particularly in operational environments, it is a training requirement for military aircrew. Similar to biomonitoring in operational environments, measurement of ScO_2_, in addition to SpO_2_, may provide more accurate thresholds that impair brain function to prevent compromising training aims. Implementing more realistic training approaches to better simulate the operational environment and provide immediate objective feedback should also be prioritised; however, any cognitive tasks integrated within the training should not supersede training objectives. The use of HRT modalities (e.g., normobaric vs. hypobaric, mask on vs. mask off) and hypoxic doses should align with training objectives since generalised training profiles may not be translatable to real-world events. If not, training may be misleading and cause additional safety risks if exact replication of hypoxic symptoms is expected. HRT will never be an exact replication of real-world hypoxia events, particularly in normobaric modalities that do not simulate the hypoxic dysbaric physiological state experienced in a depressurised aircraft. Hence, if aircrews feel abnormal during high-altitude flight, then hypoxia should always be suspected.

## Author Contributions

DS wrote the first draft of the manuscript. DS, NG, and GC wrote sections of the manuscript. All authors contributed to the article and approved the submitted version.

### Conflict of Interest

The authors declare that the research was conducted in the absence of any commercial or financial relationships that could be construed as a potential conflict of interest.
